# How does the quality of outcomes framework impact on the quality of life of adult patients with diabetes? A cross-section study in England using GP Patient Survey data

**DOI:** 10.1186/s12875-025-02930-x

**Published:** 2025-10-28

**Authors:** Mimi Xiao, Alberto Núñez-Elvira, Søren Rud Kristensen

**Affiliations:** 1 School of Public Health, Research Center for Medical and Social Development, Chongqing Medical University, Chongqing, China; 2 Department of Economics, School of Business and Economics, Universidad CEU San Pablo, Madrid, Spain; 3 Centre for Health Policy, Institute of Global Health Innovation Imperial College London, London, UK; 4 Danish Centre for Health Economics (DaCHE), University of Southern Denmark, Odense, Denmark

**Keywords:** Quality outcome framework, Quality of life, EQ-5D, Achievement score

## Abstract

**Background:**

Existing studies on the assessment of the Quality and Outcomes Framework (QOF) have found that the QOF affects clinical outcomes and quality of care. However, there is little evidence of its impact on health-related quality of life (HQoL).

**Aim:**

To investigate the association between QOF and HQoL through the assessment of different measures. First, the percentage of overall achievement scores and the five clinical domains were based on EQ-5D-5L. Second, the percentage of achievement scores and individual domains of HQoL.

**Design and setting:**

The analysis linked the national GP Patient Survey (GPPS) with the performance score in the QOF for three years (2015-/16-2017/18).

**Methods:**

Patient-level analysis was conducted on HQoL using the GPPS and the percentage of achievement scores from the QOF. We then regressed multilevel models on patient-level estimates. Finally, we estimated the impact of QOF on the individual dimensions of the EQ-5D-5L using ordered logit regression models.

**Results:**

From a total sample size of 3,489,691patients (44% male), our findings convey that patients in a practice with 1% increase in the overall achievement percentage have 0.022 (95% CI = 0.00530, 0.0386) points higher EQ-5D utility scores than those in a practice without performance improvement. Moreover, a 1% increase in the achievement percentage on diabetes mellitus (DMpc) was associated with a 0.0167 (95% CI = 0.00445, 0.0289) point increase in diabetic patients’ EQ-5D, and a positive association was observed for chronic obstructive pulmonary disease (COPD). Furthermore, patients who experienced an improvement in performance were more likely to report a better health state in one of the five dimensions of the EQ-5D (anxiety) than those in practices without performance improvement.

**Conclusion:**

QOF was associated with a limited, but positive and statistically significant impact on patients’ HRQoL.

## Introduction

After two decades of experimentation with pay-for-performance (P4P) in health care yielding mixed results [1], there is a call for a fundamental rethink of the use of financial incentives to improve the quality of care [2]. This is also true for one of the largest incentive schemes in primary care, the English Quality and Outcomes Framework (QOF) [3]. Initially, the QOF was designed to reward general practices for providing good quality of care to patients in the (NHS) [4, 5] but also to motivate GPs and increase funding for their practices as a source of extra income. Although the scheme has developed over time, it retains its core design and focuses on enhancing quality of care. Quality is measured across broad domains, of which the clinical domain is the largest. For each domain, quality is measured against a set of indicators that are made publicly available. The basis of performance payments is determined by a scoring system in which practices receive points according to their performance in achieving those indicators. QOF has been often criticised for “being restrictive in reporting domains and its limited ability to evaluate important decisions of quality of care.” [4] However, several studies show that the removal of QOF financial incentives can cause reductions in recorded quality of care for many QOF-related indicators [6] but the size of the effect seems to depend on the QOF exception rates [7].

Initially the QOF was successful in reducing variation in care and improving process quality [8]. Yet, more recently, it has been argued that the focus on process quality means that the QOF fails to incentivise holistic person-centred care which is needed for patients with complex long-term conditions [5, 9]. This view is supported by research showing that the QOF often takes over the agenda of GP consultations in a way that makes it difficult for patients to get their own priorities heard [10]. 

Strikingly, though, there is very little knowledge about the association between QOF performance and patients’ health-related quality of life. A recent systematic review of the role of the QOF in the care of long-term conditions did not identify any evidence on the impact of the QOF on patients’ quality of life, or satisfaction [11]. 

We aim to address this gap in the literature by investigating the association between practices’ QOF performance and diabetes patients’ health-related quality of life (HQoL). Previous research has focused on estimating the impact of QOF on the incentivised measures or has used mortality as a summary indicator [11]. Here, we focus on providing evidence on whether QOF incentivises the holistic perspective on patients’ wellbeing and assess how QOF performance covaries with patients’ self-reported health status.

## Methods

### Data

Our analysis links patient-level data from the General Practice Patient Survey (GPPS), which includes questions about patients’ HQoL, and practice-level data from the QOF using the practice identifier, which is available in both datasets. Our analysis focused on patients with diabetes over three years (2015/16–2017/18). Regarding missing data, we handled the missing covariates using a complete-case analysis.

### Measuring health-related quality of life

The GPPS is a large-scale England-wide survey on patients’ experiences of primary care. The survey is conducted by Ipsos MORI on behalf of NHS England. The practices in the GPPS are representative of all practices in England. Patients were included in the survey if they had a valid NHS number, registered with a GP practice continuously for at least six months before being included, and were 18 years of age or older.

Health-related quality of life was measured using the EQ-5D-5L set of questions on patients’ health states included in the GP Patient Survey (GPPS). The EQ-5D-5L is a standardised instrument developed by the EuRoQol group and has been widely used in the literature [12, 13] as a measure of HQoL for diabetic patients. It is a generic measure of health status with five dimensions: mobility, self-care, usual activities, pain/discomfort, and anxiety/depression. Each dimension has five levels: no problems (coded as 5), slight problems (4), moderate problems (3), severe problems (2) and unable (1). By combining these five dimensions, a patient’s health profile can be expressed as a single string. For example, the health profile 55555 reflects a full health state. Each health state can be converted to a utility score by attaching social preference weights to the health state elicited from the general population [14]. In addition to HQoL, GPPS contains information on patients’ age, gender, ethnicity, employment status and deprivation level of the area where the patient lives.

### Measuring quality in QOF

We collected data on practices’ overall QOF achievement rate, defined as the percentage of maximum points achieved. In addition, we gathered information on performance data related to five clinical domains relevant to patients with diabetes: diabetes mellitus (DM), coronary heart disease (CHD), stroke (STIA), chronic obstructive pulmonary disease (COPD), and hypertension (HYA). For each domain, quality was measured on a range of performance indicators such as “the percentage of patients with diabetes, on the register, with a record of a foot examination and risk classification”. The QOF data were gathered from the official website of the Quality and Outcomes Framework in NHS England Digital [15]. 

### Data linkage

**Fig. 1:**
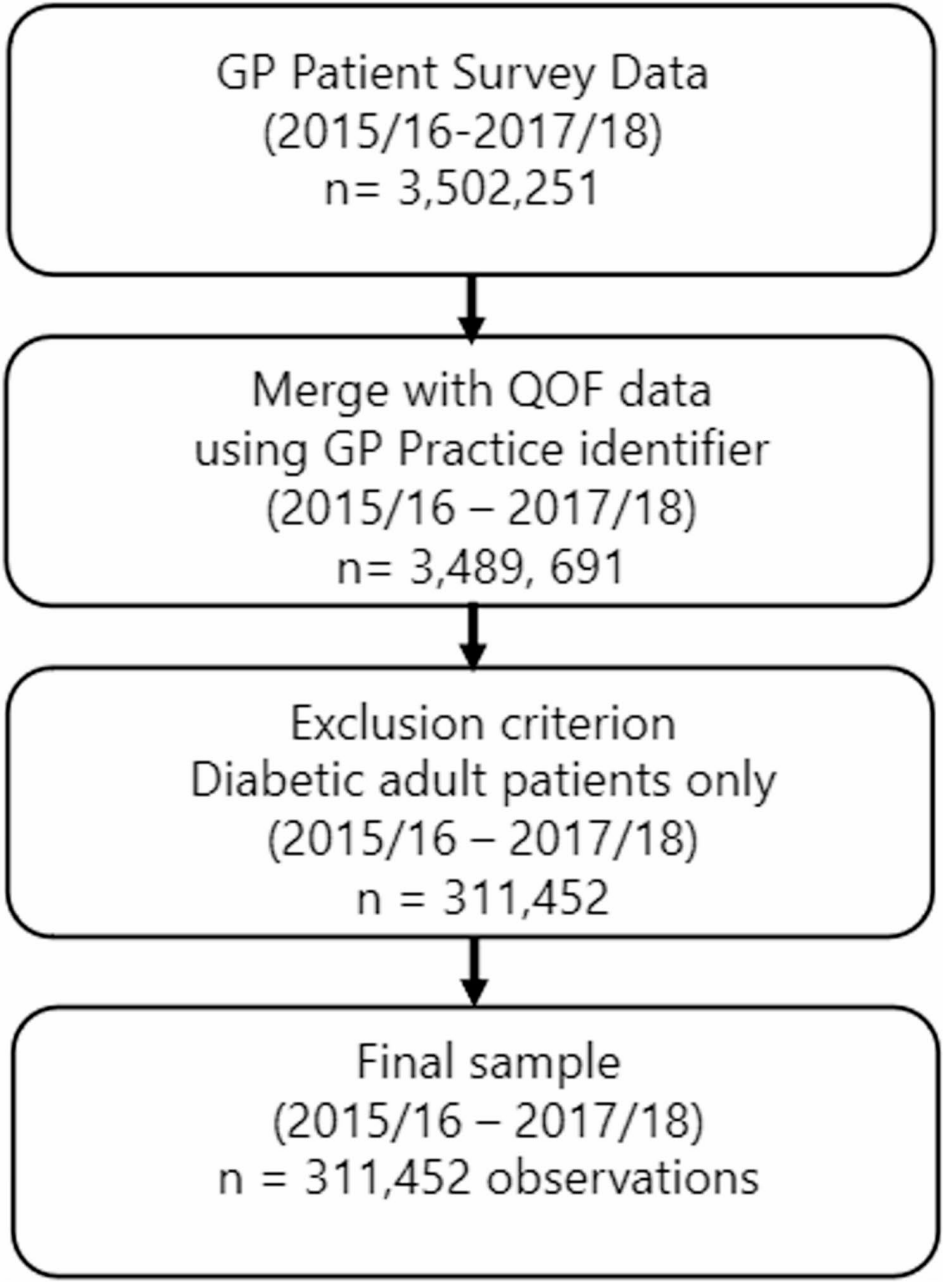
A flowchart on the merging of GPPS and QOF datasets

We built a pooled cross-section dataset. For each fiscal year between 2015/16 and 2017/18, we merged the GPPS and QOF datasets collected around June of the corresponding fiscal year, using the practice identifier present in both datasets. For instance, our 2017/18 data were obtained by merging the 2017 GPPS data with the 2017/18 QOF data on clinical domains. The GPPS 2017 data^1^ were obtained in June 2017, corresponding to the period between 2017 and 2018 when the QOF data for 2017/18 were recorded. In the sample, patients and practices were observed repeatedly, averaging 203 patients per practice. Our exclusion criteria were age and being diabetic, as we only included diabetic adults aged over 18 years. Our total sample size comprised 3,489,691 patient-year observations from 7,624 practices. A flowchart Fig 1 illustrates the merging of the GPPS and QOF datasets for each fiscal year using a GP identifier, including exclusion criteria and matching rates.

### Statistical analysis

The goal of our analysis was to estimate the association between practice QOF performance and patients’ HQoL. In our sample, the patients are nested within the practices. Therefore, we estimated multilevel models grouping patients within practices [16–20]. As an alternative, we estimated fixed effects models and a hybrid model that includes practice mean achievement and deviation from the practice mean in the hierarchical model, the so-called Random Effects Within Between (REWB) model [21] which bridges the two modelling approaches.

Our dependent variable was the patient’s quality of life which was obtained from the EQ-5D-5L utility scores, these scores are continuous variables calculated from discrete health states. The key explanatory variable of interest was the overall QOF achievement score of the patients’ practices. We adjusted for patients’ characteristics using eight-year age bands, gender, ethnicity, local area deprivation, and year fixed effects as covariates. To examine whether practice performance on individual clinical domains was associated with HQoL, we estimated models with each of the individual clinical achievement scores as explanatory variables. In addition, we explored the impact of QOF on the probability of having a better health state in the individual dimensions of the EQ-5D using an ordered logit model.

## Results

Table 1 summarises the main descriptive statistics of the variables used in the analysis. The average EQ-5D score across all patients was 0.8, the average practice achievement score was 95.95%, and the average achievement scores in the clinical domains ranged from 91.36% (diabetes mellitus) to 97.82% (stroke). Approximately 83% of patients were aged between 35 and 84 years. Most patients were white (88.1%), and 49% of the patients in the sample were employed either part-time (13.7%) or full-time (35.3%). The income deprivation scores were stratified into five quintiles across the sample.

**Table 1:** The descriptive statistics of variables in 2015-17 data

Covariates, mean (SD)	*N* = 3,489,691
EQ_5D_5L	0.8 (0.232)
Total achievement percent	95.95(6.85)
Diabetes Mellitus percent	91.36(10.78)
Coronary Heart Disease achievement percent	96.11(7.36)
Stroke achievement percent	97.82(5.45)
Chronic Obstructive Pulmonary Disease achievement percent	96.77(9.05)
Hypertension achievement percent	97.75(6.60)
Gender, male, %	44.2(49.7)
Age group	
18 to 24, %	4(19.6)
25 to 34, %	9.4(29.2)
35 to 44, %	12.8(33.4)
45 to 54, %	17.8(38.3)
55 to 64, %	20.4(40.3)
65 to 74, %	20.7(40.5)
75 to 84, %	11.3(31.7)
85 or above, %	3.5(18.3)
Ethnic group	
White, %	88.1(32.3)
mixed, %	0.8(8.9)
Asian, %	6.3(24.2)
Black, %	2.5(15.7)
Others, %	2.3(14.8)
IMD	
1 st quintile, %	20.9(40.7)
2nd quintile, %	20.6(40.4)
3rd quintile, %	20.2(40.1)
4th quintile, %	19.6(39.7)
5th quintile (Least deprived), %	18.8(39)
Employment status	
Full time work, %	35.3(47.8)
Part time work, %	13.7(34.4)
Full time educated, %	1.6(12.4)
Unemployed, %	3.5(18.4)
Sick/retired/others, %	45.9(49.8)
Number of patients per practice	203.348(50.75)

### The relationship between practice QOF performance and health related quality of life

Table 2 reports the association between the QOF performance scores of practice in individual domains and health-related quality of life. The analysis was also adjusted for covariates such as age group, gender, ethnicity, employment status, and year. To investigate the socioeconomic dimension, we incorporated deprivation indices (IMD) into the analysis to examine the effects of QOF performance on QoL for each IMD quintile. For this purpose, we added an interaction term between the achievement scores and IMD quintiles in the analysis. The results suggested that a 1% increase in the achievement percentage on diabetes mellitus (DMpc) was associated with 0.0167 (95% CI = 0.00445, 0.0289) points increase in patients’ EQ-5D-5L, a 1% increase in chronic obstructive pulmonary disease achievement (COPDpc) was associated with 0.0155 (95% CI = 0.00103, 0.0300) points increase in the EQ-5D of patients with COPD. With respect to the total percentage of achievement score, a 1% in total percentage of achievement score was associated with a 0.022 (95% CI = 0.00530, 0.0386) increase in the EQ-5D-5L of patients. The estimates for the interaction term between the achievement score and IMD quintiles showed that the relation between the QOF performance and QoL mostly did not significantly vary by IMD quintile. Patients in practices with higher performance scores on diabetes mellitus, COPD, and the total percentage of achievement score (TOTpc) on average had higher EQ-5D scores than those in practices with average performance scores. Additionally, the EQ-5D of males was higher than that of females for patients with higher income percentiles and lower for part-time work, full-time educated, unemployed, and sick/retired/others compared to work.^2^

**Table 2:** The association between the QOF performance scores of practice and the health related quality of life on sample of diabetic patients

	(1)	(2)	(3)	(4)	(5)	(6)
Dependent variable: Eq. 5D						
	Diabetes Mellitus percentage (DMpc)	Coronary Heart Diseases percentage (CHDpc)	Stroke percentage (STIApc)	Chronic Obstructive Pulmonary Disease percentage (COPDpc)	Hypertension percentage (HYApc)	Total percentage of achievement score (TOTpc)
	0.0167**	0.00225	0.00465	0.0155*	0.00166	0.0220**
	[0.00445,0.0289]	[−0.0144,0.0189]	[−0.0171,0.0264]	[0.00103,0.0300]	[−0.0165,0.0198]	[0.00530,0.0386]
Constant	0.930***	0.943***	0.941***	0.931***	0.944***	0.923***
	[0.909,0.952]	[0.919,0.967]	[0.913,0.969]	[0.907,0.954]	[0.918,0.969]	[0.899,0.948]
ICC	0.0246	0.0247	0.0247	0 0.0246	0.0247	0.0246
N	311,452	311,421	311,379	311,368	311,441	311,505

Notes: Diabetes Mellitus percentage (DM), Coronary Heart Disease percentage (CHD), Stroke percentage (STIA), Chronic Obstructive Pulmonary Disease percentage (COPD), Total percentage of achievement score (TOTpc); “95% confidence intervals in brackets. Standard errors are shown in parentheses. Control variables are not shown: gender, age groups, ethnicity groups, IMD groups (local area deprivation), employment status and year dummies *p < 0.05 **p < 0.01 ***p < 0.001

As a robustness test, we estimated the models using fixed effects and present these estimates along with estimates from Random Effects Within Between (REWB) model that uses both within and between practice variations that are reported in the Appendix 1. Although the Hausman test did not support the use of the hierarchical model, our preferred model remains this model because of the very limited within-practice variation in QOF performance, which means that the fixed effects model only uses a restricted amount of variation in the data. While QOF achievement was never statistically significant in the fixed-effects models, in all instances where the achievement score was statistically significant in the standard hierarchical model, the practice mean achievement score was also statistically significant in the REWB models.

The impact of the QOF may vary across the five dimensions of the EQ-5D. We explored the impact of QOF on the individual dimensions of EQ-5D using an ordered logit model and present the results in Table 3. A higher value for the individual dimensions was interpreted as a better health outcome. The significantly positive coefficient of the performance variable on one of the five dimensions of the EQ-5D showed that patients who benefited from the improvement in their practice performance were more likely to report a better health state on the anxiety dimension of the EQ-5D than those who were in practices without performance improvement.

**Table 3:** The impact of QOF on the indices of individual dimensions of EQ-5D

	(1)	(2)	(3)	(4)	(5)
	Mobility	Self-care	Usual activity	Pain	Anxiety
The percent of total achievement score (TOTpc)	0.0832	0.0930	0.0421	0.0916	0.127^*^
	[−0.0271,0.194]	[−0.0485,0.235]	[−0.0779,0.162]	[−0.0215,0.205]	[0.000749,0.253]
	(0.056)	(0.0722)	(0.0612)	(0.0577)	(0.0643)
N	335,159	333,642	334,692	335,097	326,641
Number of practices	7,625	7,626	7,626	7,626	7,626

Notes: Standard errors were adjusted for 7624 practice–indicator clusters. Control variables are not shown: gender, age groups, ethnicity groups, IMD groups (local area deprivation), employment status, and year dummies. 95% confidence intervals are shown in parentheses. *p < 0.05 **p < 0.01 ***p < 0.001

## Discussion

### Summary

Across the three years (2015/16–2017/18) considered in our study, our findings showed that the QOF indicator for practice performance was a statistically significantly associated with patients’ HQoL. Among the five clinical domains analysed, the performance percentage of patients with diabetes mellitus and COPD had a statistically significant positive effect on patients’ HQoL. The rationale behind these results could be that diabetes and COPD were among the original conditions prioritized by the QOF, which consistently awarded high points to these conditions and, therefore, higher income for general practices. This incentivizes practices to focus on these conditions and ensure their quality of care improves [22]. Additionally, diabetes and COPD have more standardized and measurable aspects of care, such as glycaemic control, blood pressure, and respiratory function, making it easier to assess the impact of the QOF on these aspects [23]. Moreover, the QOF targets, such as HbA1c levels in diabetes, can be directly related to the quality of life of patients. For example, achieving better glycaemic control has been shown to be associated with better clinical outcomes, and potentially, enhanced quality of life [24]. Examining the effects of QOF on the composition of EQ-5D (composition effects), our results show that performance improvement had statistically significantly positive effects on the anxiety dimension of EQ-5D. One explanation could be that the QOF performance improvement might have specifically targeted at improving mental health aspects, potentially influencing the anxiety dimension more than others [25]. Furthermore, the EQ-5D anxiety dimension might be more sensitive to changes in mental health than other dimensions, such as mobility or pain [26]. This provides initial insights into the effects of QOF on the composition of the EQ-5D. These findings suggest that QOF performance might exert a certain impact on patients’ quality of life and a small positive impact on incentivising holistic patient-centred care for patients with chronic long-term conditions.

### Strengths and limitations

The availability of our linked data by merging GPPS with QOF data at the corresponding time is a significant strength of this study. The size of the estimates suggests that the impact of experiencing QOF is not substantial in magnitude but is statistically significant. Our study findings on the impact of QOF on quality of life could be limited by confounding factors. Patients who are more likely to be in a practice with improved performance may be more capable of improving their health status. In addition, as mentioned earlier, the conventional employment of multiple regression techniques treats the units of analysis as independent observations and fails to recognise the fact that there are repeated patient observations at the practice level. The use of multilevel models could help address this limitation by including residuals at patient and practice levels. A further limitation is our inability to rule out reverse causality, i.e. that patients’ health-related quality of life may affect their ability to make the behavioural choices that could affect the QOF achievement score.

### Comparison with existing literature

There is little empirical evidence on how patients’ quality of life is affected by the QOF, and previous literature has mainly focused on the impact of the QOF on clinical outcomes or other measures of quality of care. In a study on the cost effectiveness evaluation of a pay-for-performance (P4P) program for diabetic conditions under the National Health Insurance in Taiwan [27] Tan et al. (2014) examined the influence of the program on the patients’ health-related quality of life via matching equivalent groups of patients receiving regular care, they found that the P4P program had an increase of 0.08 in QALYs (measured by SF36 health instrument) [27]. In our study, we found that the scheme had a statistically significant effect on the HQoL, which has rarely been studied.

### Implications for research and/or practice

A number of studies have been performed to evaluate the effectiveness of financial incentives on quality of care [28, 29]. This advances the debate and provides new evidence regarding the impact of financial incentives on patients’ quality of life and may be important for practitioners and policymakers. Our findings show a statistically significant association of QOF, though small, on the health-related quality of life for patients with long-term conditions which might support the current debate towards the development of a more outcomes-focused framework. In practice, incentive schemes should be tailored to patient needs. These findings are consistent with the ongoing QOF reform on the shift focus from process-based targets to patient outcomes and a greater emphasis on long-term conditions, including chronic disease management (e.g. diabetes, hypertension) and early intervention [4, 30]. It aligns with NHS Long Term Plan priorities (e.g., prevention, health inequalities).

In addition, our results have implications for policymakers considering investing in performance schemes to improve performance standards. Existing studies that conducted cost-effectiveness evaluations of performance schemes focused only on evaluating the benefits of the scheme by identifying potential benefits for patients. However, most studies underestimated other potential benefits from a societal perspective. Current studies that estimate process-based outcomes or population health, such as mortality, do not include the full societal benefits of QOF.

## Conclusions

The multi-level analysis of the QOF on EQ-5D suggested that the QOF had a significant positive effect on the EQ-5D utility score, 1% of the total performance score was associated with a 0.022 (95% CI = 0.0053, 0.0386) increase in the EQ-5D of patients and a positive association with diabetes mellitus (DMpc), and chronic obstructive pulmonary disease. Furthermore, patients who experienced improvement in performance were more likely to report a better health state in one of the five dimensions of the EQ-5D (the anxiety dimension) than those who were in practices without performance improvement.

### How this fits in

The impact of the Quality and Outcomes Framework (QOF) on health-related quality of life (HQoL) has not yet been studied. This study found new evidence on the association between the QOF scheme and improvements in the HQoL of patients and the individual dimensions of the measure.

## Data Availability

The data that support the findings of this study are available fromIpsos MORI on behalf of NHS Englandbut restrictions apply to the availability of these data, which were used under license for the current study, and are not publicly available. Data are available from the corresponding author upon reasonable request, and with permission of Ipsos MORI on behalf of NHS England (https://www.england.nhs.uk/statistics/statistical-work-areas/gp-patient-survey/). The microdata are not publicly available and can only be accessed with permission of Ipsos on behalf of NHS England. To access the data, a license is required. We confirm that NHS England or Ipsos granted us access to GPPS. The survey has been published elsewhere, the reference to it in the manuscript is GP Patient Survey (GPPS). Please see the questionnaire source (https://gp-patient.co.uk/downloads/2024/qandletter/GPPS%202024%20Questionnaire_PUBLIC.pdf).

## Appendix 1

**Table 4:** Table A1: The fixed effects estimates along with estimates from a Random Effects Within Between model

		Achivement		Achivement deviation		Mean achievement	
Diabetes Mellitus percent (DMpc)	Mixed	0.0167**	[0.00445,0.0289]				
	Fixed	−0.0077	[−0.0294,0.0140]				
	REWB			−0.00832	[−0.0297,0.0131]	0.0288***	[0.0143,0.0433]
Coronary Heart Diseases percent (CHDpc)	Mixed	0.00225	[−0.0144,0.0189]				
	Fixed	−0.0038	[−0.0308,0.0232]				
	REWB			−0.00348	[−0.0300,0.0231]	0.0061	[−0.0150,0.0272]
Stroke percent (STIApc)	Mixed	0.00465	[−0.0171,0.0264]				
	Fixed	0.00291	[−0.0326,0.0384]				
	REWB			0.00329	[−0.0319,0.0384]	0.00553	[−0.0224,0.0334]
Chronic Obstructive Pulmonary Disease percent (COPDpc)	Mixed	0.0155*	[0.00103,0.0300]				
	Fixed	0.00166	[−0.0165,0.0198]				
	REWB			−0.00458	[−0.0243,0.0151]	0.0337**	[0.0128,0.0545]
Hypertension percent (HYApc)	Mixed	0.00166	[−0.0165,0.0198]				
	Fixed	−0.0117	[−0.0405,0.0171]				
	REWB			−0.0118	[−0.0401,0.0166]	0.012	[−0.0111,0.0351]
Total percent of achievement score (TOTpc)	Mixed	0.0220**	[0.00530,0.0386]				
	Fixed	0.00632	[−0.0155,0.0282]				
	REWB			0.00753	[−0.0140,0.0290]	0.0427**	[0.0170,0.0684]

## Appendix 2

**Table 5:** Table A2: The association between the QOF performance scores of practice and the health related quality of life on full sample

	(1)	(2)	(3)	(4)	(5)	(6)
Dependent variable: Eq. 5D						
	Diabetes Mellitus percent (DMpc)	Coronary Heart Diseases percent (CHDpc)	Stroke percent (STIApc)	Chronic Obstructive Pulmonary Disease percent (COPDpc)	Hypertension percent (HYApc)	Total percent of achievement score (TOTpc)
	0.0127***	0.00783	0.0198**	0.00854*	0.00267	0.0155**
	[0.00567, 0.0198]	[−0.00192, 0.0176]	[0.00623, 0.0334]	[0.000522, 0.0166]	[−0.00785, 0.0132]	[0.00604, 0.0250]
	(0.00360)	(0.00498)	(0.00694)	(0.00409)	(0.00537)	(0.00483)
_cons	0.947***	0.951***	0.939***	0.950***	0.956***	0.943***
	[0.940, 0.953]	[0.941, 0.960]	[0.925, 0.952]	[0.942, 0.958]	[0.945, 0.966]	[0.934, 0.953]
	(0.00345)	(0.00488)	(0.00686)	(0.00408)	(0.00533)	(0.00474)
N	3,489,691	3,489,691	3,489,691	3,489,691	3,489,691	3,489,691

Diabetes Mellitus percent (DM), Coronary Heart Disease percent (CHD), Stroke percent (STIA), Chronic Obstructive Pulmonary Disease percent (COPD), Total percent of achievement score (TOTpc); “95% confidence intervals in brackets. Standard error in parentheses. Control variables not shown: gender, age groups, ethnicity groups, IMD groups (local area deprivation), employment status and year dummies

**p* < 0.05

***p* < 0.01

****p* < 0.001

## Footnotes

1 In the GPPS named as Y11.

2 The full estimates of the interaction terms are provided on request.
